# e-Learning for Instruction and to Improve Reproducibility of Scoring Tumor-Stroma Ratio in Colon Carcinoma: Performance and Reproducibility Assessment in the UNITED Study

**DOI:** 10.2196/19408

**Published:** 2021-03-19

**Authors:** Marloes A Smit, Gabi W van Pelt, Elisabeth MC Dequeker, Raed Al Dieri, Rob AEM Tollenaar, J Han JM van Krieken, Wilma E Mesker, UNITED Group

**Affiliations:** 1 Department of Surgery Leiden University Medical Center Leiden Netherlands; 2 Department of Public Health and Primary Care Biomedical Quality Assurance Research Unit University of Leuven Leuven Belgium; 3 European Society of Pathology Brussels Belgium; 4 Department of Pathology Radboud University Medical Center Nijmegen Netherlands

**Keywords:** colon cancer, tumor-stroma ratio, validation, e-Learning, reproducibility study, cancer, tumor, colon, reproducibility, carcinoma, prognosis, diagnostic, implementation, online learning

## Abstract

**Background:**

The amount of stroma in the primary tumor is an important prognostic parameter. The tumor-stroma ratio (TSR) was previously validated by international research groups as a robust parameter with good interobserver agreement.

**Objective:**

The Uniform Noting for International Application of the Tumor-Stroma Ratio as an Easy Diagnostic Tool (UNITED) study was developed to bring the TSR to clinical implementation. As part of the study, an e-Learning module was constructed to confirm the reproducibility of scoring the TSR after proper instruction.

**Methods:**

The e-Learning module consists of an autoinstruction for TSR determination (instruction video or written protocol) and three sets of 40 cases (training, test, and repetition sets). Scoring the TSR is performed on hematoxylin and eosin–stained sections and takes only 1-2 minutes. Cases are considered stroma-low if the amount of stroma is ≤50%, whereas a stroma-high case is defined as >50% stroma. Inter- and intraobserver agreements were determined based on the Cohen κ score after each set to evaluate the reproducibility.

**Results:**

Pathologists and pathology residents (N=63) with special interest in colorectal cancer participated in the e-Learning. Forty-nine participants started the e-Learning and 31 (63%) finished the whole cycle (3 sets). A significant improvement was observed from the training set to the test set; the median κ score improved from 0.72 to 0.77 (*P*=.002).

**Conclusions:**

e-Learning is an effective method to instruct pathologists and pathology residents for scoring the TSR. The reliability of scoring improved from the training to the test set and did not fall back with the repetition set, confirming the reproducibility of the TSR scoring method.

**Trial Registration:**

The Netherlands Trial Registry NTR7270; https://www.trialregister.nl/trial/7072

**International Registered Report Identifier (IRRID):**

RR2-10.2196/13464

## Introduction

The prediction of disease outcome for an individual patient as part of personalized medicine is becoming routine practice in the management of cancer patient treatment. Staging of colon cancer by pathologists is based on hematoxylin and eosin (H&E)-stained sections of the primary tumor. The tumor-node-metastasis (TNM) classification is used as the main selection criterion for additional treatment, along with noting of characteristics such as depth of invasion and differentiation grade [1], according to the American Joint Committee staging algorithm. However, conventional H&E sections provide more information than previously recognized.

In the last decade, research has not only focused on the tumor and its characteristics but increasingly also on the tumor microenvironment. The tumor microenvironment consists of the stromal background with a variety of cells such as fibroblasts, endothelial cells, and lymphocytes. The tumor-stroma ratio (TSR) is the amount of tumor relative to the amount of stroma in the primary tumor [2-4]. Patients with a high amount of stroma (stroma-high) have a worse prognosis compared to those harboring tumors with a low amount of stroma (stroma-low) in multiple types of cancer [5-13].

Scoring the TSR is performed on H&E-stained sections in only 1-2 minutes, with good to excellent interobserver agreement [3]. This implies that TSR scoring is easy to learn and useful in daily practice.

The Uniform Noting for International Application of the Tumor-Stroma Ratio as an Easy Diagnostic Tool (UNITED) study was developed to prepare for implementation of the TSR as an additional high-risk indicator along with traditional TNM classification. As part of the UNITED study, an instruction protocol and reproducibility study were initiated using an e-Learning module as described in the published research protocol [14].

Digital pathology is increasingly being implemented in daily diagnostic practice as well as for teaching. Digital pathology for instruction, most commonly used for instruction of students, has multiple advantages: more students can be reached because it is web-based; all students look at exactly the same case; annotations can be shared with the teacher, resulting in direct feedback; and students can complete the course when and where it suits them [15-18].

The use of e-Learning for education has been adopted in different medical specialties worldwide. An example of e-Learning used in pathology is a module developed for Dutch pathologists [19,20]. The module focuses on decreasing the variation in grading dysplasia in adenomas and increasing the consistency of scoring serrated lesions. Two separate studies have shown that e-Learning is a good method to improve performance [19,20].

e-Learning to instruct professionals has also been confirmed in specialties other than pathology [21,22]. For instance, based on a systematic review for surgical training (students, residents, and surgeons), Maertens et al [23] concluded that e-Learning is as effective as other methods for training.

The aim of this study was to confirm the high reproducibility of scoring the TSR using an e-Learning module to train a variety of pathologists.

## Methods

### Case Selection

The e-Learning module was based on H&E-stained sections of stage II and III colon cancer resection specimens. The cases were randomly selected from the archives of the Pathology Department of Leiden University Medical Centre (LUMC). The number of cases with very low stroma (ie, 10% or 20% stroma area) were limited from the analysis to increase the number of cases that are generally more difficult to score for pathologists and are therefore more suitable for training purposes. In the e-Learning, 55% of the cases were stroma-low (≤50% stroma) and 45% were stroma-high (>50% stroma). None of the patients had received neoadjuvant treatment at the time of sample collection. The sample size was based on a workable amount of cases to maintain quality without the case load being too high.

Slides were scanned using a 20× objective with the Panoramic 250 scanner (3D Histech) or with the IntelliSite Digital pathology slide scanner (Philips).

### Participants

Pathologists and pathology residents from all over the world could participate in the UNITED study and in the e-Learning. The UNITED study started in 2018, and pathologists were invited to start (and complete) the e-Learning in the period of December 2017 to April 2019. Data collection ended in April 2019. Multimedia Appendix 1 provides an overview of participating countries, and the numbers of participating pathologists and residents.

### TSR Scoring Method

The previously published protocol for scoring the TSR was used in this study [3,4]. In brief, the section of the deepest part of the tumor, usually the section used for determining the T-stage, was chosen. The area with the highest amount of stroma was selected and scored at 100× magnification in increments of 10%. A field should contain tumor cells on four opposite edges of the field of evaluation.

### e-Learning

The e-Learning module was developed in PathXL Tutor version 6.1.1.1. (Philips, Belfast, UK). This software uses digital images, and was developed to easily share and teach a network of pathology students, or in this case pathologists. The PathXL software allows the pathologist to analyze the slide in a manner comparable to using a microscope. The e-Learning was prepared to resemble real-life microscopy as far as possible by using round annotations with a fixed size of 3.4 mm^2^. This represents the size of the field of vision of microscopes, even from different brands, when using 100× magnification.

Participants were blinded to clinical data of the sections and were only informed that the patients did not receive neoadjuvant treatment.

Before starting the first set of the e-Learning module, participants were asked to watch the instruction video on the study website [24]. The training set consisted of 40 cases. This set started with 5 multiple choice questions where annotations were placed upfront in different areas of the section (Multimedia Appendix 2). Participants were asked to select the correctly placed annotation and determine the percentage of stroma. In the other 35 cases (and in the other sets), the participant was asked to place the annotations themselves at the most optimal position and to determine the stroma percentage.

The test set also consisted of 40 cases, including 37 (93%) new cases. To determine the intraobserver agreement of scoring the TSR, the test set was repeated (repetition set) after a 2-month washout period with the same cases placed in a different order.

The answer model used for evaluating the results was established by two experienced observers (GP, MS) together with a pathologist (VS) of the LUMC. The coordinators of the UNITED study checked all finished e-Learning sets for stroma percentage and for correct placement of the annotation. The answers of the participants were compared with the answer model. Continuing with the second set was allowed when an interobserver agreement (κ) of ≥0.7 with predefined scores was reached. In the case in which a participant did not pass a set due to a κ score below 0.7, the same set had to be rescored (see Multimedia Appendix 3 for the flowchart of the e-Learning module [14]) after feedback was given.

### Statistical Analysis

Data collected in this study comprised: (1) whether the participant is a pathologist or resident, (2) participating country, (3) the stroma percentage of the different questions, and (4) whether or not the participant considered a question to be difficult. In this study, a possible bias could be that participating pathologists/residents are generally more motivated for participation.

Stroma percentages were classified as stroma-low (≤50% stroma) or as stroma-high (>50% stroma) [3,4,25]. This dichotomous output and the outcome of whether or not the annotation was placed correctly were used for measuring observer agreement. Cohen κ coefficient was used to measure inter- and intraobserver agreement. This score is quantitative and was used as a noncontinuous variable. Histograms were used to visualize the distribution of the κ scores for each set.

Nonnormally distributed continuous variables are described with the median and range (minimum and maximum values). For the median κ scores of a set, the first κ score of a participant of each set was used. The Wilcoxon signed-rank test was used to compare paired nonnormally distributed continuous variables (eg, measuring the progress between different e-Learning sets by participants).

### Ethical Considerations

The UNITED study protocol has been approved by the Medical Research Ethics Committee of the LUMC (study number p17.302). All samples were handled in accordance with the 1964 Helsinki Declaration and its later amendments or comparable ethical standards.

## Results

### Participants

**Figure 1:**
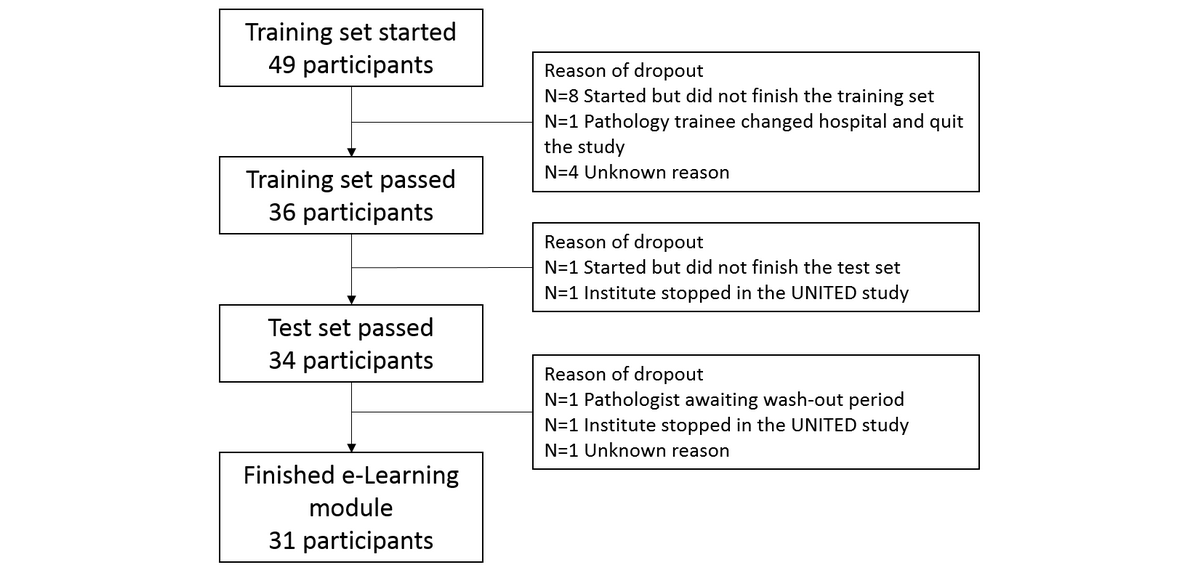
Overview of the number of participants of the e-Learning and the reasons for not finishing the e-Learning cycle.

In total, 63 participants (49 pathologists and 14 residents) were registered for e-Learning. However, 14 participants were nonresponsive after registration, and thus 36 pathologists and 13 residents from 19 countries started the e-Learning; these 49 participants were used for analysis. All participating pathologists had gastrointestinal pathology as a subspecialty; however, most of them had more than one subspecialty. The residents had not yet chosen a pathology subspecialty. Thirty-six (73%) participants (28 pathologists and 8 residents) finished the training set and continued with the test set. In total, 31 (63%) participants finished the whole cycle (3 sets) of the e-Learning. A complete overview and reasons for participants to drop out are shown in Figure 1. The participants who quit the study were left out of the analysis.

### Observer Agreement

**Figure 2:**
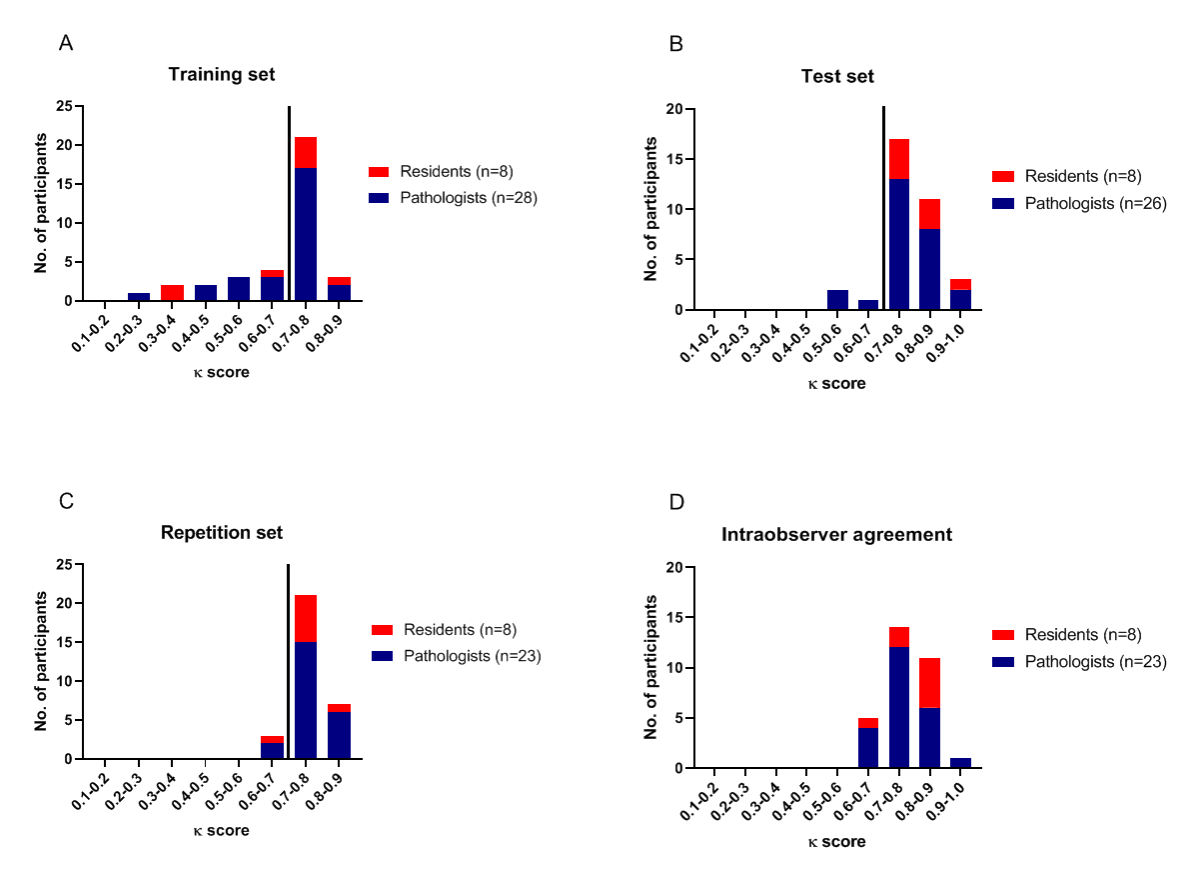
Distribution of κ scores for each set of the e-Learning: (A) training set, (B) test set, (C) repetition set, and (D) intraobserver agreement.

**Figure 3:**
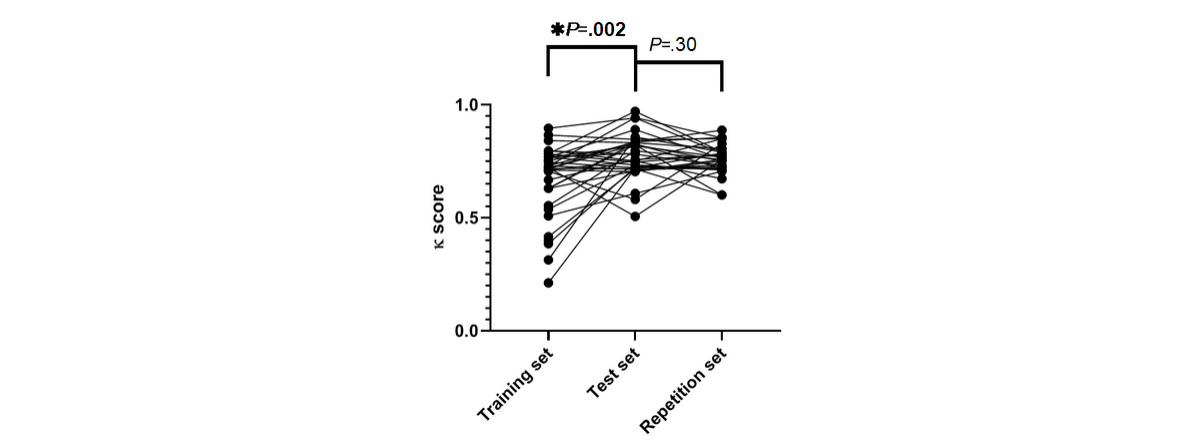
Progress of κ scores per participant during the e-Learning module.

After finishing the training set of the e-Learning, the observer agreement was determined. Twenty-four (67%) participants (19 pathologists and 5 residents) passed the training set at the first attempt (ie, κ≥0.7). Two participants (both residents) needed a third chance to pass the training set. The median κ score for the training set was 0.72. The test set was passed the first time by 31 (91%) participants (23 pathologists and 8 residents) and 3 (9%) pathologists had to repeat the test set. The median κ score for the test set was 0.77. After a 2-month washout period, 28 (90%) participants (21 pathologists and 7 residents) directly passed the repetition set, with a median κ score of 0.76 (Table 1, Figure 2). A significant improvement was observed from the training set to the test set (*P*=.002). No significant changes of the κ scores were observed between the test set and the repetition set (*P*=.30, Figure 3). Intraobserver agreement was measured for the 31 participants who finished the repetition set. The median κ score of the intraobserver agreement was 0.77 (Table 1). Scoring results from pathologists showed significant improvement from the training set to the test set (*P*=.006) and no fall back (*P*=.74) after a washout period of 2 months. The scoring results of the residents showed no significant changes between sets (*P*=.26 and *P*=.13). Details are shown in Multimedia Appendix 4.

**Table 1:** Distribution of κ scores per set.

Set	Completed set, N	κ, median (range)
Training set	36	0.72 (0.21-0.90)
Test set	34	0.77 (0.51-0.97)
Repetition set	31	0.76 (0.60-0.89)
Intraobserver agreement	31	0.77 (0.61-0.95)

### Difficulty of the Questions

For each case, participants were asked whether scoring the TSR of the section was easy, normal, or difficult. If answered as difficult, a reason could be given. A case was classified as difficult when at least 40% of the participants agreed with this assessment. Eleven cases (9 different cases) were classified as difficult (4 in the training set, 2 in the test set, and 5 in the repetition set). The cases classified as difficult in the test set remained difficult in the repetition set. The three other cases of the repetition set were more difficult than average in the test set. As expected, most of the difficult cases were those close to the cut-off value of 50%, mucinous tumors, tumors with a lot of necrosis, and tumors in which the distinction between the stroma and the smooth muscle was difficult (see Multimedia Appendix 5 for examples of difficult cases). Overall, the 11 cases were more often answered wrong (29% of the answers) by participants who classified (one of) these cases as difficult compared to 19% of wrong answers for cases that were assessed as not difficult (see Multimedia Appendix 6 for the subdivision per case).

### Drawing Annotations

**Figure 4:**
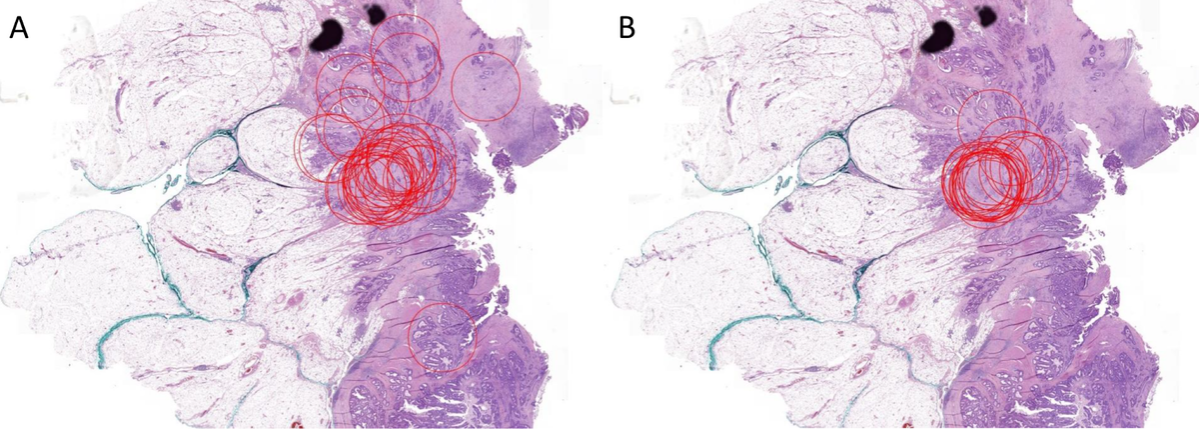
Example of improvement of selecting the most optimal area for scoring the tumor-stroma ratio. Diffuse spread of annotations was seen in the training set (A) compared to less variation of the scoring area in the test set (B).

**Figure 5:**
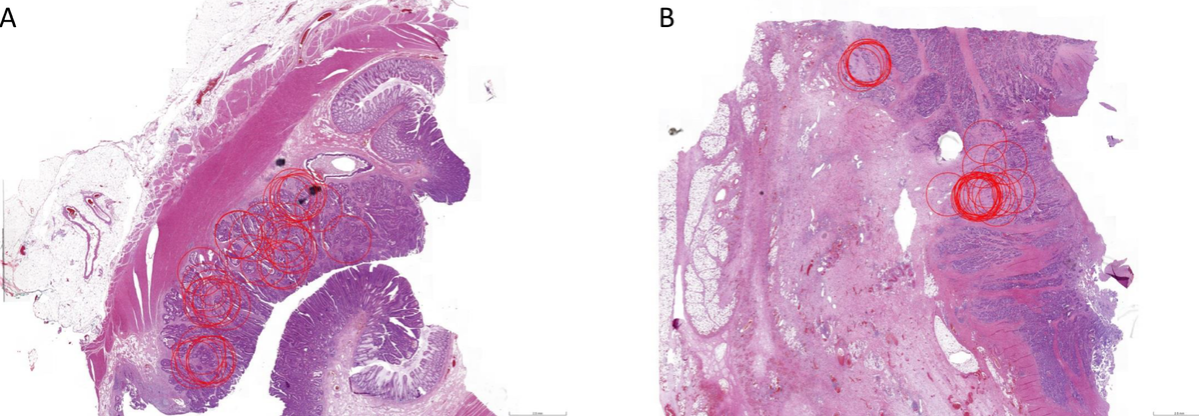
Examples of the distribution of annotations. In stroma-low cases (A), a wide distribution was more frequent, whereas in stroma-high cases, a hotspot (or sometimes more) formed (B).

A few cases were used in both the training and test sets. Analysis of these cases showed progress of scoring at the hotspot, as the annotations were more centered (Figure 4). Furthermore, in stroma-low cases, annotations were more widely spread over the entire tumor area, whereas one or two hotspots were more often identified in stroma-high cases (Figure 5).

## Discussion

This study shows that e-Learning is an accurate method to instruct pathologists and pathology residents for scoring the TSR. A κ score >0.7 was used to define the reproducibility of the method. The median κ score improved and the minimum value increased after completing the consecutive sets for scoring the TSR. Significant progress (*P*=.002) was observed from the training to the test set. If a set was not passed the first time, feedback was given to the participant before they repeated the same set; however, participants did not get insight into their precise mistakes. The feedback was personalized, but not case-specific.

A decrease was observed between the number of created accounts, the number of participants who started the e-Learning, and the number of participants who completed the whole module. There are multiple reasons for this decrease. Most of the participants were registered by the principal investigator of an institute; however, not everyone responded to their registration. Another reason for dropout was withdrawal of the center from the UNITED study.

In daily diagnostics, H&E-stained sections are used for determining cancer stage. To ensure the high quality of the sections used for e-Learning, the original H&E-stained slides of the cases used for diagnostic purposes were scanned. Furthermore, all slides were scanned at the same magnification to avoid differences in the quality of the digital images.

When reviewing the e-Learning sets, in some cases it seemed as if the participants had scored the tumor percentage instead of the stroma percentage. This might be explained by the fact that pathologists are accustomed to scoring the neoplastic cell percentage for molecular analysis. Another explanation might be more related to semantics. The amount of tumor is in the numerator, which might be a source of confusion. In these doubtful cases, the participant was asked to reevaluate the case as well as some others. Thus, scoring the percentage of stroma remains a point of attention.

Overall, participants were well able to choose the right area for scoring the stroma percentage and to estimate whether a section was stroma-high or stroma-low. A common misinterpretation was scoring at the invasive front instead of looking for an area with as much stroma as possible within the section. This might be explained by the fact that pathologists are accustomed to scoring the tumor budding at the invasive front [26]. Furthermore, as the scoring protocol describes the use of the section from the deepest part of the tumor (usually the section used for determining the T-status) [3], the distinction between scoring the TSR in the whole tumor area and not necessarily at the invasive front might not have been made clear enough in the instructions. When comparing the results of pathologists and residents, pathologists showed significant improvement from the training to the test set, whereas residents did not. A possible explanation is the small group of residents (n=8) or the fact that pathologists are more experienced than residents.

Research performed on other pathology biomarkers used in daily practice such as lymphovascular invasion [27], tumor grading [28,29], classification and grading of colorectal polyps or adenomas [19,20,30-33], and the estimation of tumor cell percentage [34] has shown weak to moderate interobserver agreement. With three median κ scores above 0.7, the interobserver agreement for scoring the TSR in this study was found to be good. Although no comparison arm was included in this study, the median κ values obtained in this study are lower than those reported previously. This can be explained by the fact that the scores were low in the training set, which improved in the test set.

Digital pathology is increasingly entering pathology practice, although most pathology departments are not yet (fully) digitalized. In the future, a digital image analysis program will be useful for more accurate scoring and even better reproducibility. Digital pathology for teaching goals has some advantages and disadvantages. The advantages include easy distribution of samples and being able to score a set at the same time while reaching a worldwide group of pathologists, and disadvantages include possible software flaws or bugs when using a digitized workflow. In this study, the placed annotations were not always saved correctly, which sometimes made it difficult to analyze the results for a participant. In these particular cases, the participant was asked to reevaluate the case.

In conclusion, this study showed that e-Learning is a good and effective method to instruct pathologists and residents in scoring the TSR. Furthermore, this study confirmed the reproducibility of the scoring method.

## Appendix

Multimedia Appendix 1 Overview of the participating countries and number of participating pathologists or residents per country.

Multimedia Appendix 2 Example of a multiple choice question of the e-Learning. Each participant was asked to select the annotation at the most optimal position following the criteria mentioned in the scoring protocol.

Multimedia Appendix 3 Flowchart for the instruction of participating pathologists using the e-Learning module.

Multimedia Appendix 4 Progress of κ scores per participant separated for pathologists (A) and residents (B).

Multimedia Appendix 5 Examples of difficult cases for scoring the tumor-stroma ratio (TSR) as mentioned by the participants. (A) A case around the cut off-value of 50%. (B) Necrosis makes it difficult to select the most optimal site for the annotation. (C) A mucinous tumor can sometimes be difficult to score, because mucus has to be visually excluded from the scorings area. (D) Stromal tissue and smooth muscle tissue.

Multimedia Appendix 6 Subdivision of the 11 questions classified as difficult, showing the proportion of wrong answers per question.

## Abbreviations

H&E: hematoxylin and eosin
LUMC: Leiden University Medical Centre
TNM: tumor node metastasis
TSR: tumor-stroma ratio
UNITED: Uniform Noting for International Application of the Tumor-Stroma Ratio as an Easy Diagnostic Tool

## References

[1] Labianca R, Nordlinger B, Beretta G, Mosconi S, Mandalà M, Cervantes A, Arnold D, ESMO Guidelines Working Group (2013). Early colon cancer: ESMO Clinical Practice Guidelines for diagnosis, treatment and follow-up. Ann Oncol.

[2] Mesker WE, Liefers G, Junggeburt JMC, van Pelt GW, Alberici P, Kuppen PJK, Miranda NF, van Leeuwen KAM, Morreau H, Szuhai K, Tollenaar RAEM, Tanke HJ (2009). Presence of a high amount of stroma and downregulation of SMAD4 predict for worse survival for stage I-II colon cancer patients. Cell Oncol.

[3] van Pelt GW, Kjær-Frifeldt S, van Krieken JHJM, Al Dieri R, Morreau H, Tollenaar RAEM, Sørensen FB, Mesker WE (2018). Scoring the tumor-stroma ratio in colon cancer: procedure and recommendations. Virchows Arch.

[4] Mesker WE, Junggeburt JMC, Szuhai K, de Heer P, Morreau H, Tanke HJ, Tollenaar RAEM (2007). The carcinoma-stromal ratio of colon carcinoma is an independent factor for survival compared to lymph node status and tumor stage. Cell Oncol.

[5] Wu J, Liang C, Chen M, Su W (2016). Association between tumor-stroma ratio and prognosis in solid tumor patients: a systematic review and meta-analysis. Oncotarget.

[6] Zhang R, Song W, Wang K, Zou S (2017). Tumor-stroma ratio (TSR) as a potential novel predictor of prognosis in digestive system cancers: A meta-analysis. Clin Chim Acta.

[7] Kramer CJH, Vangangelt KMH, van Pelt GW, Dekker TJA, Tollenaar RAEM, Mesker WE (2019). The prognostic value of tumour-stroma ratio in primary breast cancer with special attention to triple-negative tumours: a review. Breast Cancer Res Treat.

[8] Wang K, Ma W, Wang J, Yu L, Zhang X, Wang Z, Tan B, Wang N, Bai B, Yang S, Liu H, Zhu S, Cheng Y (2012). Tumor-stroma ratio is an independent predictor for survival in esophageal squamous cell carcinoma. J Thorac Oncol.

[9] Dekker TJA, van de Velde CJH, van Pelt GW, Kroep JR, Julien J, Smit VTHBM, Tollenaar RAEM, Mesker WE (2013). Prognostic significance of the tumor-stroma ratio: validation study in node-negative premenopausal breast cancer patients from the EORTC perioperative chemotherapy (POP) trial (10854). Breast Cancer Res Treat.

[10] Eriksen AC, Sørensen FB, Lindebjerg J, Hager H, dePont Christensen R, Kjær-Frifeldt S, Hansen TF (2018). The prognostic value of tumour stroma ratio and tumour budding in stage II colon cancer. A nationwide population-based study. Int J Colorectal Dis.

[11] Peng C, Liu J, Yang G, Li Y (2018). The tumor-stromal ratio as a strong prognosticator for advanced gastric cancer patients: proposal of a new TSNM staging system. J Gastroenterol.

[12] Kemi N, Eskuri M, Herva A, Leppänen J, Huhta H, Helminen O, Saarnio J, Karttunen TJ, Kauppila JH (2018). Tumour-stroma ratio and prognosis in gastric adenocarcinoma. Br J Cancer.

[13] Pongsuvareeyakul T, Khunamornpong S, Settakorn J, Sukpan K, Suprasert P, Intaraphet S, Siriaunkgul S (2015). Prognostic evaluation of tumor-stroma ratio in patients with early stage cervical adenocarcinoma treated by surgery. Asian Pac J Cancer Prev.

[14] Smit M, van Pelt G, Roodvoets A, Meershoek-Klein Kranenbarg E, Putter H, Tollenaar R, van Krieken JH, Mesker W (2019). Uniform noting for international application of the tumor-stroma ratio as an easy diagnostic tool: protocol for a multicenter prospective cohort study. JMIR Res Protoc.

[15] Parker EU, Reder NP, Glasser D, Henriksen J, Kilgore MR, Rendi MH (2017). NDER: a novel web application for teaching histology to medical students. Acad Pathol.

[16] Helle L, Nivala M, Kronqvist P, Gegenfurtner A, Björk P, Säljö R (2011). Traditional microscopy instruction versus process-oriented virtual microscopy instruction: a naturalistic experiment with control group. Diagn Pathol.

[17] Sahota M, Leung B, Dowdell S, Velan GM (2016). Learning pathology using collaborative vs. individual annotation of whole slide images: a mixed methods trial. BMC Med Educ.

[18] Samulski TD, Taylor LA, La T, Mehr CR, McGrath CM, Wu RI (2018). The utility of adaptive eLearning in cervical cytopathology education. Cancer Cytopathol.

[19] IJspeert JEG, Madani A, Overbeek LIH, Dekker E, Nagtegaal ID (2017). Implementation of an e-learning module improves consistency in the histopathological diagnosis of sessile serrated lesions within a nationwide population screening programme. Histopathology.

[20] Madani A, Kuijpers CCHJ, Sluijter CE, Von der Thüsen JH, Grünberg K, Lemmens VEPP, Overbeek LIH, Nagtegaal ID (2019). Decrease of variation in the grading of dysplasia in colorectal adenomas with a national e-learning module. Histopathology.

[21] Nakanishi H, Doyama H, Ishikawa H, Uedo N, Gotoda T, Kato M, Nagao S, Nagami Y, Aoyagi H, Imagawa A, Kodaira J, Mitsui S, Kobayashi N, Muto M, Takatori H, Abe T, Tsujii M, Watari J, Ishiyama S, Oda I, Ono H, Kaneko K, Yokoi C, Ueo T, Uchita K, Matsumoto K, Kanesaka T, Morita Y, Katsuki S, Nishikawa J, Inamura K, Kinjo T, Yamamoto K, Yoshimura D, Araki H, Kashida H, Hosokawa A, Mori H, Yamashita H, Motohashi O, Kobayashi K, Hirayama M, Kobayashi H, Endo M, Yamano H, Murakami K, Koike T, Hirasawa K, Miyaoka Y, Hamamoto H, Hikichi T, Hanabata N, Shimoda R, Hori S, Sato T, Kodashima S, Okada H, Mannami T, Yamamoto S, Niwa Y, Yashima K, Tanabe S, Satoh H, Sasaki F, Yamazato T, Ikeda Y, Nishisaki H, Nakagawa M, Matsuda A, Tamura F, Nishiyama H, Arita K, Kawasaki K, Hoppo K, Oka M, Ishihara S, Mukasa M, Minamino H, Yao K (2017). Evaluation of an e-learning system for diagnosis of gastric lesions using magnifying narrow-band imaging: a multicenter randomized controlled study. Endoscopy.

[22] Canty D, Barth J, Yang Y, Peters N, Palmer A, Royse A, Royse C (2019). Comparison of learning outcomes for teaching focused cardiac ultrasound to physicians: A supervised human model course versus an eLearning guided self- directed simulator course. J Crit Care.

[23] Maertens H, Madani A, Landry T, Vermassen F, Van Herzeele I, Aggarwal R (2016). Systematic review of e-learning for surgical training. Br J Surg.

[24] Tumor-stroma ratio. UNITED.

[25] Huijbers A, Tollenaar R, v Pelt G, Zeestraten E, Dutton S, McConkey C, Domingo E, Smit V, Midgley R, Warren B, Johnstone E, Kerr D, Mesker W (2013). The proportion of tumor-stroma as a strong prognosticator for stage II and III colon cancer patients: validation in the VICTOR trial. Ann Oncol.

[26] Lugli A, Kirsch R, Ajioka Y, Bosman F, Cathomas G, Dawson H, El Zimaity H, Fléjou JF, Hansen TP, Hartmann A, Kakar S, Langner C, Nagtegaal I, Puppa G, Riddell R, Ristimäki A, Sheahan K, Smyrk T, Sugihara K, Terris B, Ueno H, Vieth M, Zlobec I, Quirke P (2017). Recommendations for reporting tumor budding in colorectal cancer based on the International Tumor Budding Consensus Conference (ITBCC) 2016. Mod Pathol.

[27] Harris E, Lewin D, Wang H, Lauwers G, Srivastava A, Shyr Y, Shakhtour B, Revetta F, Washington M (2008). Lymphovascular invasion in colorectal cancer: an interobserver variability study. Am J Surg Pathol.

[28] Chandler I, Houlston RS (2008). Interobserver agreement in grading of colorectal cancers-findings from a nationwide web-based survey of histopathologists. Histopathology.

[29] Barresi V, Reggiani Bonetti L, Ieni A, Branca G, Tuccari G (2016). Histologic prognostic markers in stage IIA colorectal cancer: a comparative study. Scand J Gastroenterol.

[30] Jensen P, Krogsgaard MR, Christiansen J, Braendstrup O, Johansen A, Olsen J (1995). Observer variability in the assessment of type and dysplasia of colorectal adenomas, analyzed using kappa statistics. Dis Colon Rectum.

[31] Terry MB, Neugut AI, Bostick RM, Potter JD, Haile RW, Fenoglio-Preiser CM (2002). Reliability in the classification of advanced colorectal adenomas. Cancer Epidemiol Biomarkers Prev.

[32] Denis B, Peters C, Chapelain C, Kleinclaus I, Fricker A, Wild R, Augé B, Gendre I, Perrin P, Chatelain D, Fléjou JF (2009). Diagnostic accuracy of community pathologists in the interpretation of colorectal polyps. Eur J Gastroenterol Hepatol.

[33] Costantini M, Sciallero S, Giannini A, Gatteschi B, Rinaldi P, Lanzanova G, Bonelli L, Casetti T, Bertinelli E, Giuliani O, Castiglione G, Mantellini P, Naldoni C, Bruzzi P, SMAC Workgroup (2003). Interobserver agreement in the histologic diagnosis of colorectal polyps. the experience of the multicenter adenoma colorectal study (SMAC). J Clin Epidemiol.

[34] Smits AJJ, Kummer JA, de Bruin PC, Bol M, van den Tweel JG, Seldenrijk KA, Willems SM, Offerhaus GJA, de Weger RA, van Diest PJ, Vink A (2014). The estimation of tumor cell percentage for molecular testing by pathologists is not accurate. Mod Pathol.

