# Supplementary material for: e-Learning for Instruction and to Improve Reproducibility of Scoring Tumor-Stroma Ratio in Colon Carcinoma: Performance and Reproducibility Assessment in the UNITED Study
Source: JMIR Form Res. 2021 Mar 19;5(3):e19408. doi: 10.2196/19408 (PMC8122297; doi:10.2196/19408)
Supplement: Multimedia Appendix 1 [file formative_v5i3e19408_app1.pdf]

e-Learning for instruction and to improve reproducibility of scoring Tumor-Stroma Ratio in Colon Carcinoma: Performance and Reproducibility Assessment in the UNITED Study. Marloes A Smit et al.

Corresponding author: W.E. Mesker (w.e.mesker@lumc.nl)

Multimedia Appendix 1 Overview of the participating countries and number of participating pathologists or residents per country.

Country	No. of pathologists	No. of residents	Total
Austria	1	0	1
Brazil	2	0	2
Croatia	1	0	1
Czech Republic	1	3	4
Danmark	1	1	2
France	1	0	1
Germany	1	0	1
Iceland	1	0	1
Macedonia	5	2	7
Netherlands	3	0	3
Portugal	1	0	1
Serbia	2	1	3
Slovenia	1	0	1
Spain	1	1	2
Ukraine	2	0	2
United Kingdom	4	0	4
Total	28	8	36
