# Supplementary material for: e-Learning for Instruction and to Improve Reproducibility of Scoring Tumor-Stroma Ratio in Colon Carcinoma: Performance and Reproducibility Assessment in the UNITED Study
Source: JMIR Form Res. 2021 Mar 19;5(3):e19408. doi: 10.2196/19408 (PMC8122297; doi:10.2196/19408)
Supplement: Multimedia Appendix 3 [file formative_v5i3e19408_app3.pdf]

e-Learning for instruction and to improve reproducibility of scoring Tumor-Stroma Ratio in Colon Carcinoma: Performance and Reproducibility Assessment in the UNITED Study. Marloes A Smit et al.

Corresponding author: W.E. Mesker (w.e.mesker@lumc.nl)

Multimedia Appendix 3 Flowchart for the instruction of participating pathologists using the e-Learning module.

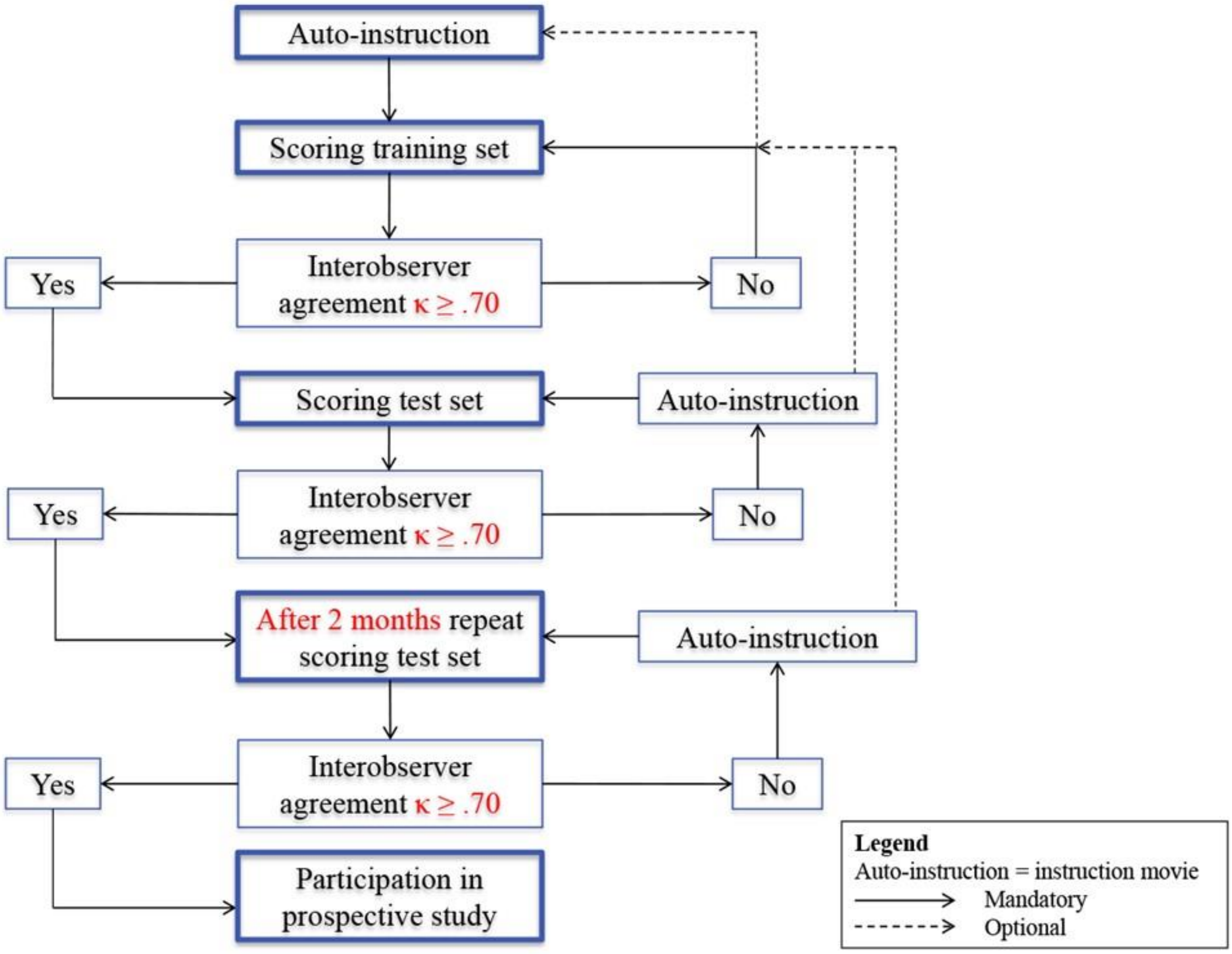

From: *Smit M, et al.* Uniform Noting for International Application of the Tumor-Stroma Ratio as an Easy Diagnostic Tool: Protocol for a Multicenter Prospective Cohort Study. JMIR Res Protoc 2019;8(6):e13464.
