# Supplementary material for: e-Learning for Instruction and to Improve Reproducibility of Scoring Tumor-Stroma Ratio in Colon Carcinoma: Performance and Reproducibility Assessment in the UNITED Study
Source: JMIR Form Res. 2021 Mar 19;5(3):e19408. doi: 10.2196/19408 (PMC8122297; doi:10.2196/19408)

e-Learning for instruction and to improve reproducibility of scoring Tumor-Stroma Ratio in Colon Carcinoma: Performance and Reproducibility Assessment in the UNITED Study. Marloes A Smit et al. Corresponding author: W.E. Mesker (w.e.mesker@lumc.nl)

Multimedia Appendix 4 Progress of κ scores per participant separated for pathologists (A) and residents (B).

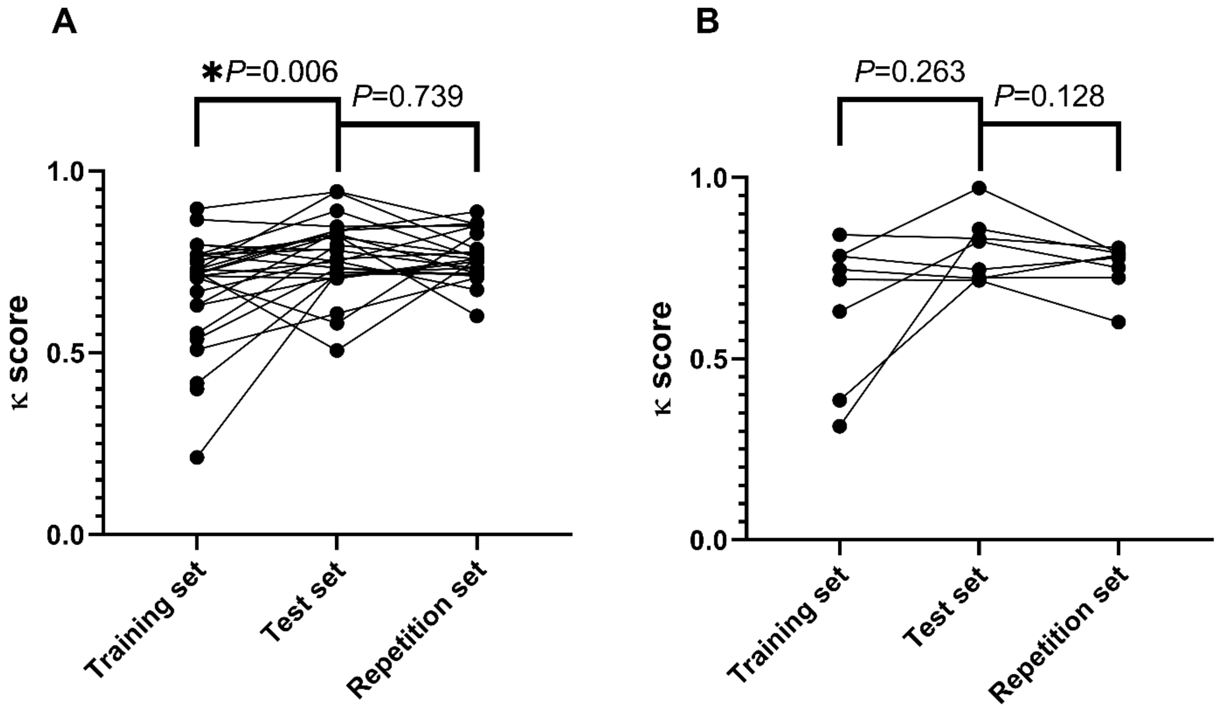

Supplement: Multimedia Appendix 4 [file formative_v5i3e19408_app4.pdf]
