# Supplementary material for: e-Learning for Instruction and to Improve Reproducibility of Scoring Tumor-Stroma Ratio in Colon Carcinoma: Performance and Reproducibility Assessment in the UNITED Study
Source: JMIR Form Res. 2021 Mar 19;5(3):e19408. doi: 10.2196/19408 (PMC8122297; doi:10.2196/19408)

e-Learning for instruction and to improve reproducibility of scoring Tumor-Stroma Ratio in Colon Carcinoma: Performance and Reproducibility Assessment in the UNITED Study. Marloes A Smit et al. Corresponding author: W.E. Mesker (w.e.mesker@lumc.nl)

Multimedia Appendix 5 Examples of difficult cases for scoring the tumor-stroma ratio (TSR) as mentioned by the participants: A) a case around the cut off value of 50%, B) necrosis makes it difficult to select the most optimal place for the annotation, C) a mucinous tumor can sometimes be difficult because mucus has to be visually excluded from the scorings area, D) stromal tissue and smooth muscle tissue.

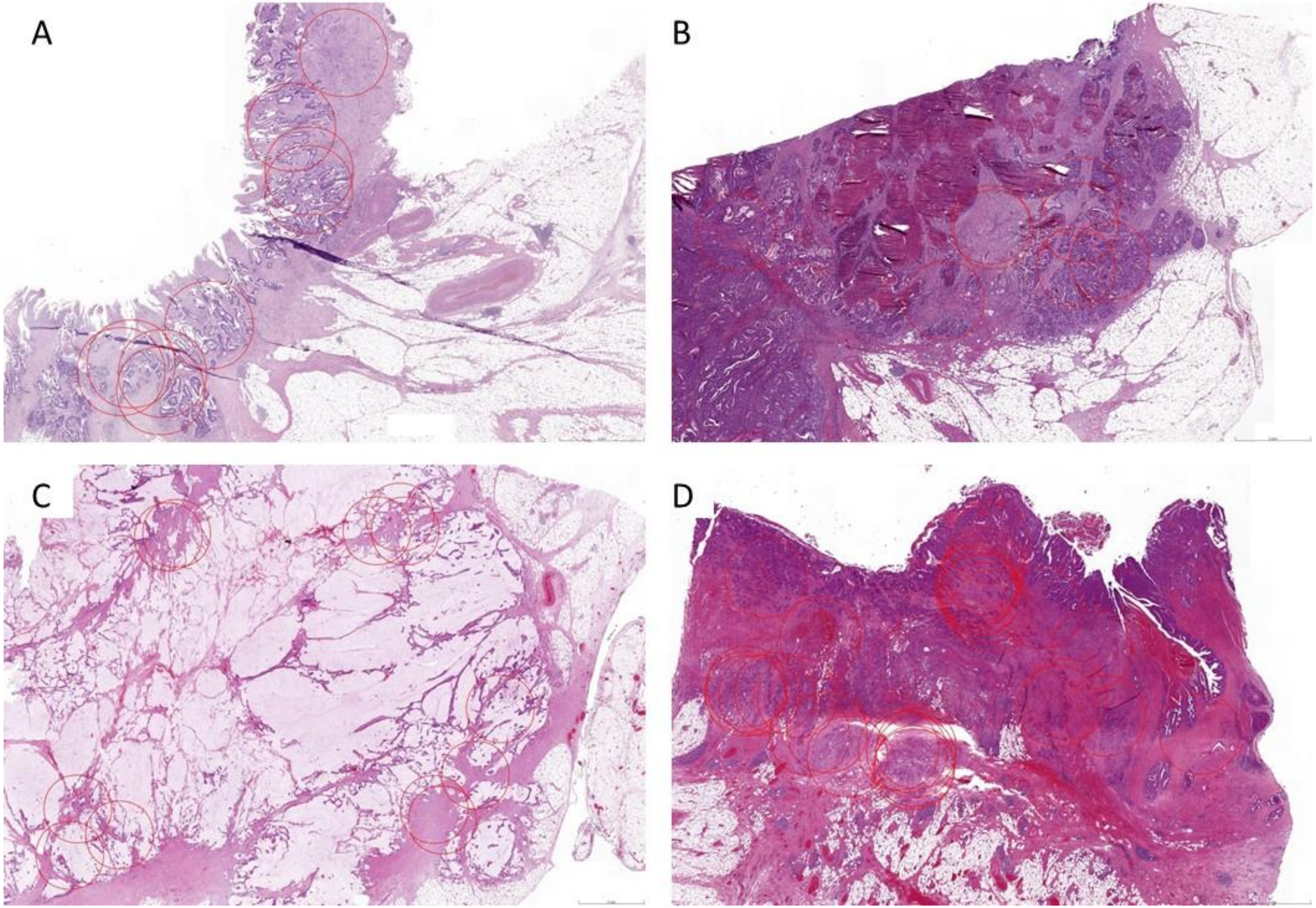

Supplement: Multimedia Appendix 5 [file formative_v5i3e19408_app5.pdf]
