# Supplementary material for: e-Learning for Instruction and to Improve Reproducibility of Scoring Tumor-Stroma Ratio in Colon Carcinoma: Performance and Reproducibility Assessment in the UNITED Study
Source: JMIR Form Res. 2021 Mar 19;5(3):e19408. doi: 10.2196/19408 (PMC8122297; doi:10.2196/19408)
Supplement: Multimedia Appendix 6 [file formative_v5i3e19408_app6.pdf]

e-Learning for instruction and to improve reproducibility of scoring Tumor-Stroma Ratio in Colon Carcinoma: Performance and Reproducibility Assessment in the UNITED Study. Marloes A Smit et al.

Corresponding author: W.E. Mesker (w.e.mesker@lumc.nl)

Multimedia Appendix 6 Subdivision of the 11 questions, classified as difficult, showing the proportion of wrong answers per question.

		Difficulty			
	Case	Not difficult		Difficult	
Training set	1	2/15	13%	6/25	24%
	2	1/22	4.5%	0/18	0%
	3	5/23	22%	3/17	18%
	4	6/21	29%	1/19	5.3%
Test set	5	0/17	0%	3/17	18%
	6	6/12	50%	12/21	57%
Repetition set	7	0/14	0%	3/17	18%
	8	6/19	32%	7/12	58%
	9 (case 5)	1/17	5.9%	1/14	7%
	10 (case 6)	3/10	30%	9/21	43%
	11	5/16	31%	12/15	80%
Total		35/186	19%	56/195	29%

No. of wrong answers/total number of answers given
